# Steroid-related mechanisms in ocular health and disease: therapeutic effects, adverse events, and stress-related modulators. A narrative literature overview

**DOI:** 10.3389/fmed.2026.1877733

**Published:** 2026-07-03

**Authors:** Fabio Scarinci, Giulio Pocobelli, Francesca Romana Patacchioli

**Affiliations:** 1 Department of Ophthalmology, San Giovanni-Addolorata Hospital, Via Santo Stefano Rotondo, Rome, Italy; 2 Department of Microbiology, Immunology, Infectious Diseases, and Transplants (MIMIT), University Tor Vergata, Rome, Italy; 3 Sapienza University of Rome, Rome, Italy

**Keywords:** central serous chorioretinopathy, glaucoma, glucocorticoid therapy, neuroendocrine dysregulation, ocular health, stress

## Abstract

This narrative overview examines the interplay between stress-related neuroendocrine dysregulation, glucocorticoid signaling, and ocular health, with particular emphasis on their roles in ocular disease pathophysiology and clinical management. A structured literature search was conducted using PubMed, Scopus, and Web of Science to identify English-language peer-reviewed studies published between January 2013 and May 2025. Additional records were identified through manual screening of reference lists. Following title, abstract, and full-text evaluation according to predefined eligibility criteria, 35 studies were included in the final narrative synthesis. The available evidence indicates that activation of the hypothalamic–pituitary–adrenal axis and sympathetic nervous system influence key biological processes involved in ocular homeostasis, including intraocular pressure regulation, inflammatory signaling, vascular permeability, and neurodegenerative pathways. Stress-related neuroendocrine dysregulation has been associated with several ocular disorders, particularly glaucoma, ocular surface disease, and central serous chorioretinopathy (CSC). At the same time, glucocorticoids remain indispensable therapeutic agents for the management of inflammatory and immune-mediated ocular diseases, although their use may be accompanied by adverse effects such as ocular hypertension, glaucoma, cataract formation, delayed wound healing, and increased susceptibility to infection. The review further highlights emerging concepts related to chronobiology, advanced corticosteroid delivery systems, and personalized treatment approaches aimed at optimizing therapeutic efficacy while minimizing treatment-related complications. Collectively, the findings support an integrated model in which neuroendocrine, inflammatory, vascular, and psychosocial factors interact to influence ocular disease susceptibility, progression, and treatment outcomes. Greater recognition of stress as a modifiable contributor to ocular health may facilitate the development of more individualized and multidisciplinary approaches to ophthalmic care.

## 1 Introduction

Psychological and physical stress activate complex neuroendocrine pathways, primarily mediated by the hypothalamic–pituitary–adrenal (HPA) axis and the sympathetic–adreno-medullary system, resulting in the systemic release of glucocorticoids and catecholamines (1–3). These mediators play a central role in maintaining physiological homeostasis but may also contribute to tissue dysfunction when their regulation is altered. In the eye, sustained neuroendocrine activation has been increasingly implicated in the modulation of intraocular pressure, vascular permeability, immune responses, and neuronal integrity, thereby influencing the onset and progression of several ocular diseases (4–7).

In parallel, exogenous glucocorticoids represent a cornerstone in the management of inflammatory and immune-mediated ocular disorders, including uveitis, postoperative inflammation, retinal vascular diseases, and ocular surface conditions (8–10). Their potent anti-inflammatory and immunosuppressive properties provide substantial therapeutic benefits; however, prolonged or inappropriate exposure is associated with well-recognized adverse effects, such as intraocular pressure elevation, cataract formation, delayed wound healing, and increased susceptibility to infections (11–14). Consequently, optimizing steroid therapy requires careful consideration of dose, formulation, route of administration, and treatment duration, as well as individual patient susceptibility (8).

Importantly, endogenous stress-related glucocorticoid secretion and therapeutic steroid administration converge on shared molecular and cellular signaling pathways within ocular tissues. Disruption of these pathways may amplify inflammatory responses, alter trabecular meshwork function, impair vascular regulation, and promote neurodegenerative processes (11, 15–18). Moreover, emerging evidence indicates that circadian variations in cortisol secretion and peripheral clock regulation influence ocular physiology and treatment responsiveness, suggesting that chronobiological factors may represent an additional determinant of therapeutic efficacy and safety (4, 19, 20).

Beyond biological mechanisms, psychosocial stress and emotional distress have been shown to affect treatment adherence, disease perception, and quality of life in patients with chronic ocular conditions (5, 15, 21, 22). This bidirectional interaction between psychological burden and visual impairment may further exacerbate disease progression and compromise long-term outcomes, underscoring the need for a comprehensive, patient-centered approach to ophthalmic care.

Despite growing interest in the role of stress and glucocorticoid signaling in ocular pathology, existing evidence remains fragmented across experimental, clinical, and translational studies. Current data derive largely from observational cohorts, preclinical models, and heterogeneous clinical investigations, limiting direct comparison and generalizability (16, 23, 24). A systematic integration of these findings is therefore needed to clarify the relative contribution of endogenous neuroendocrine dysregulation and exogenous steroid exposure to disease susceptibility, progression, and therapeutic response.

Therefore, the aim of this literature review is to critically synthesize current clinical and translational evidence on the interaction between stress-related endocrine responses and glucocorticoid therapy in ophthalmology. By integrating mechanistic insights with clinical observations, this review seeks to identify key pathways underlying ocular dysfunction, highlight potential modifiable risk factors, and support the development of personalized and multidisciplinary treatment strategies to optimize patient outcomes (8, 9, 12, 17, 25).

## 2 Methods

### 2.1 Literature search strategy

This narrative review was undertaken to synthesize current evidence regarding the interplay between stress, glucocorticoid signaling, and ocular health. A structured literature search was conducted using PubMed, Scopus, and Web of Science to identify relevant studies published between January 2013 and May 2025. The final literature search was conducted in May 2025. This time frame was selected to capture contemporary advances in neuroendocrine regulation, glucocorticoid biology, ocular imaging technologies, and emerging evidence regarding stress-related ocular disorders while maintaining a clinically relevant body of literature. Seminal studies published before 2013 were considered selectively when necessary to provide historical or mechanistic context.

Boolean search strategies were applied to refine queries and improve the precision and relevance of retrieved records (26). The search strategy combined terms related to glucocorticoids, stress, and ocular disease. The PubMed search query was as follows:

(“glucocorticoids” OR “corticosteroids” OR “glucocorticoid therapy”) AND (“ocular health” OR eye OR ocular OR ophthalmic OR ophthalmology OR retinal OR choroidal) AND (stress OR “psychological stress” OR “stress-induced disease” OR “acute stress” OR “chronic stress”)

Additional keyword combinations were used to enhance specificity, including “glucocorticoid therapy” AND “ocular health,” “corticosteroids” AND (“benefits” OR “risks”) AND “eye diseases,” and (“stress-induced disease” OR “acute and chronic stress”) AND “steroids.” Equivalent search strategies adapted to database-specific indexing systems and controlled vocabularies were applied in Scopus and Web of Science. To maximize retrieval of relevant literature, the reference lists of eligible publications were manually screened for additional studies not identified through database searching.

### 2.2 Eligibility criteria and study selection

Studies were considered eligible if they were peer-reviewed articles published in English and investigated stress-related endocrine responses, glucocorticoid signaling pathways, corticosteroid therapy, or their associations with ocular physiology, pathology, or clinical outcomes. Experimental, observational, translational, and clinical studies were included to provide a comprehensive overview of the available evidence.

Editorials, letters to the editor, conference abstracts, duplicate records, non-English publications, and studies lacking direct relevance to the scope of the review were excluded. No restrictions were imposed regarding participant age, sex, ethnicity, or geographic location.

All coauthors independently screened titles and abstracts for relevance. Potentially eligible articles subsequently underwent full-text evaluation, and any disagreements regarding study eligibility were resolved through discussion until consensus was achieved.

The initial database search yielded 97 records, with an additional 16 records identified through manual screening of reference lists. Following title and abstract screening, 53 records were excluded because they were duplicates, editorials, letters, conference abstracts, non-English publications, or lacked relevance to the review topic. The remaining 60 articles underwent full-text assessment. Of these, 25 studies were excluded because they did not meet the predefined eligibility criteria or were not directly relevant to the objectives of the review. Consequently, 35 studies were included in the final narrative synthesis.

### 2.3 Data synthesis

As the objective of this work was to provide a comprehensive narrative synthesis rather than a quantitative systematic review, formal meta-analytic procedures were not performed. Relevant data were extracted independently by the authors, including study design, population characteristics, exposure to stress or glucocorticoids, principal clinical outcomes, and mechanistic findings.

Given the heterogeneity of study designs and outcome measures, findings were synthesized qualitatively and organized according to major thematic domains, including the role of corticosteroids in ocular health, stress-related ocular disorders, glucocorticoid-mediated molecular mechanisms, patient-centered considerations, and future therapeutic perspectives. Literature coverage was considered sufficient when the identified studies consistently addressed these key themes and additional screened articles did not contribute substantially novel concepts or evidence (23).

This review was conducted as a narrative overview and therefore did not formally adhere to PRISMA guidelines. Nevertheless, the review process incorporated a structured search strategy, predefined eligibility criteria, independent screening, transparent study selection procedures, and thematic synthesis to enhance methodological rigor and reproducibility (23).

## 3 Results and discussion

The available evidence highlights a complex interplay between stress-related neuroendocrine dysregulation, glucocorticoid signaling, and ocular disease. Both endogenous hormonal responses to stress and exogenous corticosteroid therapy influence key biological processes involved in ocular homeostasis, including inflammatory regulation, vascular permeability, intraocular pressure control, and neurodegeneration. Understanding these interactions is particularly relevant because glucocorticoids represent both physiological mediators of stress responses and essential therapeutic agents in ophthalmology. To facilitate interpretation of the literature, this discussion is organized into four complementary domains: (i) the role of corticosteroids in ocular health, (ii) ocular disorders associated with stress and steroid exposure, (iii) patient-centered considerations, and (iv) future perspectives. This framework provides a clinically oriented overview of how stress biology and glucocorticoid pharmacology converge in the pathophysiology and management of ocular disease.

### 3.1 Role of corticosteroids in ocular health

#### 3.1.1 Therapeutic benefits and clinical applications

Glucocorticoids remain a cornerstone in the management of inflammatory and immune-mediated ocular disorders, including uveitis, postoperative inflammation, retinal vascular diseases, and severe ocular surface conditions (8, 18). Through glucocorticoid receptor-mediated regulation of cytokine production, leukocyte migration, and vascular permeability, corticosteroids effectively suppress inflammation and contribute to the restoration of tissue homeostasis. Their efficacy, broad clinical applicability, and rapid onset of action have established corticosteroids as one of the most frequently prescribed therapeutic classes in ophthalmology.

Advances in drug formulation and delivery have further expanded the therapeutic potential of corticosteroids. Topical, periocular, intravitreal, and suprachoroidal administration routes allow treatment to be tailored according to disease location and severity. More recently, sustained-release implants and nanoparticle-based delivery systems have been developed to improve ocular bioavailability and prolong therapeutic activity while reducing systemic exposure (8, 27). Despite these advances, the benefits of corticosteroid therapy must always be balanced against the risk of treatment-related complications.

The principal therapeutic applications, benefits, and limitations of corticosteroid therapy in ophthalmology are summarized in Table 1.

**Table 1:** Clinical benefits, risks, and emerging strategies for corticosteroid use in ophthalmology.

Domain	Therapeutic benefits	Potential adverse effects	Proposed mechanisms	Key references
Intraocular Inflammation	Effective control of uveitis, retinal vasculitis, and postoperative inflammation	Steroid-induced ocular hypertension and glaucoma	Trabecular meshwork remodeling, extracellular matrix accumulation, and impaired aqueous humor outflow	(10, 11, 17)
Ocular Surface Disease	Reduction of corneal and conjunctival inflammation	Delayed epithelial healing and increased infection risk	Local immunosuppression and impaired tissue repair	(13, 14)
Retinal and Choroidal Disorders	Reduction of macular edema and inflammatory retinal disease	Increased risk of central serous chorioretinopathy (CSC) in susceptible individuals	Altered choroidal vascular permeability and glucocorticoid signaling	(32, 42)
Lens	Preservation of visual function through inflammation control	Posterior subcapsular cataract formation	Oxidative stress, epithelial cell dysfunction, and apoptosis	(12, 28)
Drug Delivery Innovations	Improved bioavailability and prolonged therapeutic activity	Long-term safety and regulatory challenges remain uncertain	Sustained-release implants, liposomal systems, and nanoparticle-based delivery platforms	(8, 27)
Personalized Treatment Strategies	Optimization of efficacy and safety through individualized care	Requires careful monitoring and risk stratification	Identification of steroid responders, dose adjustment, and steroid-sparing approaches	(18, 30, 31)

CSC, central serous chorioretinopathy.

#### 3.1.2 Mechanisms of steroid-induced ocular complications

Although highly effective, prolonged or repeated exposure to corticosteroids may induce significant ocular adverse effects. Steroid-induced ocular hypertension is among the most extensively documented complications and is primarily attributed to glucocorticoid receptor-mediated alterations within the trabecular meshwork. These changes include extracellular matrix accumulation, cytoskeletal remodeling, and impaired cellular phagocytic activity, all of which contribute to increased aqueous humor outflow resistance and elevated intraocular pressure (10, 11, 17).

At the lens level, chronic glucocorticoid exposure has been associated with oxidative stress, dysregulated epithelial cell metabolism, and apoptosis, mechanisms that contribute to posterior subcapsular cataract formation (12, 28). Corticosteroid-induced immunosuppression may also compromise local antimicrobial defenses, increasing susceptibility to infectious keratitis and delaying corneal wound healing (13, 14).

Emerging evidence suggests that excessive glucocorticoid signaling may also influence retinal and optic nerve health. Experimental studies indicate that prolonged glucocorticoid exposure can promote glial activation, inflammasome signaling, and neuroinflammatory responses that may contribute to retinal ganglion cell dysfunction and progressive optic nerve damage (17, 20, 29). These findings underscore the complex biological effects of corticosteroids, extending beyond their well-established anti-inflammatory activity.

#### 3.1.3 Clinical risk factors and monitoring strategies

The occurrence of steroid-related ocular complications is influenced by both treatment-related and patient-specific factors. Established risk factors include elevated baseline intraocular pressure, family history of glaucoma, diabetes mellitus, high myopia, connective tissue disorders, and prolonged or repeated corticosteroid exposure (10, 12, 30, 31). The route of administration also plays an important role, with periocular and intravitreal delivery generally associated with a greater risk of ocular hypertension than topical administration (18, 30).

Given this variability in individual susceptibility, careful monitoring remains essential throughout corticosteroid therapy. Regular assessment of intraocular pressure, particularly during the first months of treatment and after dose escalation, is recommended to identify steroid responders before irreversible optic nerve damage occurs. Periodic lens examination and evaluation of ocular surface integrity should also be incorporated into follow-up protocols, especially in patients requiring long-term treatment.

Collectively, these observations emphasize that the successful use of corticosteroids requires a balance between therapeutic efficacy and safety. An individualized approach that integrates risk assessment, monitoring strategies, and emerging drug-delivery technologies may help optimize outcomes while minimizing treatment-related complications.

### 3.2 Ocular disorders associated with stress and steroid exposure

Accumulating evidence indicates that stress-related neuroendocrine dysregulation contributes to the development and progression of several ocular disorders. Activation of the hypothalamic–pituitary–adrenal (HPA) axis and sympathetic nervous system results in increased cortisol and catecholamine secretion, which may influence intraocular pressure regulation, ocular surface homeostasis, choroidal circulation, and neuroinflammatory pathways (1–3, 15, 16, 19). Although the strength of evidence varies among disease entities, available data support a clinically relevant relationship between stress biology and ocular health.

The principal ocular conditions associated with stress-related mechanisms and the corresponding clinical evidence are summarized in Table 2.

**Table 2:** Stress-related ocular disorders: clinical evidence and proposed mechanisms.

Ocular condition	Clinical evidence	Proposed mechanisms	Key references
Glaucoma and increased intraocular pressure (IOP)	Acute and chronic psychological stress have been associated with transient and sustained IOP elevation, altered circadian IOP regulation, and glaucoma progression. Mindfulness-based interventions have shown beneficial effects on IOP and stress biomarkers.	HPA-axis activation, increased cortisol secretion, sympathetic nervous system stimulation, vascular dysregulation, and neuroinflammation.	(4–7, 15, 16, 21, 24, 32)
Ocular surface disease (dry eye disease)	Chronic stress is associated with increased ocular surface inflammation, tear film instability, and greater symptom severity.	NF-κB activation, pro-inflammatory T-cell polarization, disruption of ocular surface immune tolerance, and immune dysregulation.	(19, 22, 33)
Central serous chorioretinopathy (CSC)	Elevated stress levels, altered cortisol secretion, and autonomic imbalance are frequently reported in CSC patients and may contribute to disease onset, recurrence, and chronicity.	HPA-axis dysregulation, sympathetic overactivity, choroidal vascular hyperpermeability, and retinal pigment epithelium dysfunction.	(34–38, 40)
Psychosocial and behavioral factors	Anxiety, depression, and emotional distress influence disease perception, treatment adherence, follow-up compliance, and quality of life across chronic ocular disorders.	Chronic stress responses, maladaptive coping mechanisms, and behavioral influences on healthcare engagement.	(5, 19, 21, 22)

CSC, central serous chorioretinopathy; HPA, hypothalamic–pituitary–adrenal; IOP, intraocular pressure; NF-κB, nuclear factor kappa B.

#### 3.2.1 Increased intraocular pressure and glaucoma

Among stress-associated ocular disorders, glaucoma has been the most extensively investigated. Experimental studies have demonstrated that physiological circadian fluctuations in intraocular pressure are regulated through coordinated interactions between glucocorticoid signaling and sympathetic nervous system activity. Animal models show that disruption of either adrenal glucocorticoid secretion or sympathetic innervation abolishes normal IOP rhythmicity, highlighting the importance of systemic neuroendocrine regulation in aqueous humor dynamics (4).

Clinical evidence further supports this association. Patients with endogenous cortisol excess exhibit elevated IOP and altered diurnal pressure patterns, whereas individuals with adrenal insufficiency generally display lower IOP values and more physiological circadian fluctuations (32). Acute psychological stress has also been shown to induce transient IOP elevations in glaucoma patients through activation of both sympathetic and HPA-axis pathways (6, 7, 24).

Beyond acute physiological changes, chronic stress may influence long-term glaucoma progression. Persistent activation of the HPA axis can promote cortisol dysregulation, vascular dysfunction, and neuroinflammatory responses that contribute to retinal ganglion cell injury and optic nerve damage (15, 16, 20). In addition, psychological distress may negatively affect medication adherence and follow-up compliance, thereby indirectly increasing the risk of disease progression (5, 22).

Importantly, stress-reduction interventions may provide clinically meaningful benefits. A randomized controlled trial demonstrated that mindfulness-based stress reduction was associated with lower IOP, normalization of stress biomarkers, and modulation of gene-expression profiles related to inflammation and neuroprotection in patients with primary open-angle glaucoma (21).

#### 3.2.2 Ocular surface disorders

Stress-related biological mechanisms also affect ocular surface homeostasis. Experimental evidence indicates that physical and psychological stress can disrupt ocular surface integrity through activation of inflammatory signaling pathways. Desiccating stress has been shown to activate nuclear factor-κB signaling, promote T-cell polarization toward pro-inflammatory phenotypes, and induce corneal epithelial injury, ultimately contributing to tear film instability and ocular surface inflammation (33).

These findings provide mechanistic support for clinical observations linking chronic stress to increased dry eye disease severity. Neuroendocrine dysregulation may contribute to epithelial barrier dysfunction, impaired tear production, and sustained inflammatory responses.

The relationship is likely bidirectional. Persistent ocular discomfort and visual disturbance may themselves contribute to emotional distress, creating a self-perpetuating cycle in which psychological stress exacerbates ocular surface inflammation while chronic symptoms further increase psychosocial burden (19, 22).

#### 3.2.3 Central serous chorioretinopathy

Central serous chorioretinopathy (CSC) represents one of the ocular conditions most consistently associated with stress-related neuroendocrine dysregulation. Multiple studies have reported elevated stress levels, altered cortisol secretion patterns, and increased sympathetic activity in affected patients (34–37). Dysregulation of the HPA axis and sympatho-adrenal-medullary system may promote choroidal vascular hyperpermeability and retinal pigment epithelium dysfunction, ultimately leading to subretinal fluid accumulation (35–37).

The relationship between stress and CSC is likely multifactorial. While some studies suggest that psychological stress contributes to disease onset and recurrence, others indicate that visual impairment itself may generate significant emotional distress, making causality difficult to establish (19, 34). Nevertheless, growing evidence supports a contributory role of psychosocial factors in disease chronicity and recurrence (34–36).

An additional consideration is the well-established association between CSC and corticosteroid exposure. Both endogenous hypercortisolism and exogenous glucocorticoid therapy have been linked to increased CSC risk, suggesting that glucocorticoid signaling represents a shared biological pathway connecting stress physiology and steroid-related adverse effects (35, 38).

Taken together, current evidence suggests that CSC arises from a complex interaction among neuroendocrine, vascular, inflammatory, and environmental factors rather than from psychological stress alone.

### 3.3 Patient-centered considerations

#### 3.3.1 Psychosocial stress, disease perception, and treatment adherence

Beyond biological mechanisms, psychosocial factors play an important role in shaping clinical outcomes in chronic ocular disorders. Visual impairment often affects autonomy, social functioning, emotional well-being, and quality of life. Consequently, many patients experience anxiety, uncertainty, depressive symptoms, and fear of progressive vision loss, all of which may influence disease perception and treatment adherence (5, 19, 22).

Evidence from qualitative and observational studies suggests that psychological distress may reduce medication compliance, limit engagement with follow-up care, and negatively affect self-management behaviors in patients with chronic ophthalmic conditions (22).

Conversely, interventions targeting psychological well-being appear capable of improving both patient-reported outcomes and biological markers of disease. Mindfulness-based stress reduction programs have been associated with reductions in IOP, normalization of stress biomarkers, and modulation of gene-expression profiles linked to inflammation and neuroprotection (21).

#### 3.3.2 Integrated pathophysiological model

Taken together, the available evidence supports a multidimensional framework linking stress-related neuroendocrine dysregulation, glucocorticoid signaling, and ocular disease. Activation of the HPA axis and sympathetic nervous system may influence ocular tissues through interconnected inflammatory, vascular, metabolic, and neurodegenerative pathways (1–3, 15, 16, 19).

Acute stress responses generally induce transient physiological alterations, whereas chronic stress may promote persistent HPA-axis dysregulation, immune imbalance, endothelial dysfunction, and neuroinflammation, ultimately contributing to tissue remodeling and disease progression (16, 19, 39).

This integrated perspective helps explain the heterogeneous manifestations observed across glaucoma, ocular surface disease, and CSC, while emphasizing the need for multidisciplinary approaches that address both biological and psychosocial determinants of ocular health.

### 3.4 Future perspectives

#### 3.4.1 Chronobiology and optimization of steroid therapy

Growing evidence suggests that circadian biology may influence both ocular physiology and therapeutic responses. Endogenous cortisol secretion follows a tightly regulated circadian rhythm coordinated by central and peripheral biological clocks, with peak levels occurring during the early morning hours (4, 9).

Chrono-pharmacological approaches have therefore emerged as a potential strategy to optimize corticosteroid therapy. Aligning glucocorticoid administration with physiological cortisol rhythms may enhance therapeutic efficacy while minimizing adverse effects associated with excessive or prolonged glucocorticoid exposure (4, 9).

#### 3.4.2 Advances in drug delivery and nanoformulations

Recent advances in ocular drug-delivery systems seek to maximize the therapeutic benefits of corticosteroids while reducing treatment-related toxicity. Nanoparticle-based formulations, liposomal carriers, biodegradable implants, and sustained-release delivery platforms have demonstrated promising results in improving drug penetration, prolonging therapeutic activity, and reducing systemic exposure (8, 27).

These technologies may be particularly valuable for chronic inflammatory conditions requiring prolonged corticosteroid therapy.

#### 3.4.3 Personalized therapeutic approaches

Interindividual variability in glucocorticoid responsiveness represents a major challenge in ophthalmic practice. Differences in glucocorticoid receptor sensitivity, genetic background, systemic comorbidities, and environmental factors may influence both therapeutic efficacy and susceptibility to adverse effects (17, 18).

Future treatment strategies are therefore likely to move toward increasingly personalized models of care. These approaches may include individualized risk stratification, early identification of steroid responders, implementation of steroid-sparing immunomodulatory therapies, and incorporation of patient-specific psychosocial and behavioral factors into treatment planning (18, 31).

Ultimately, integrating biological, clinical, and psychosocial determinants of disease may enable the development of more effective and safer treatment paradigms. Such multidisciplinary approaches have the potential to improve both visual outcomes and quality of life while advancing the broader goal of personalized ophthalmic care.

## 4 Conclusion

This review highlights the complex interplay between stress-related neuroendocrine dysregulation and glucocorticoid signaling in the pathophysiology and management of ocular disease. Current evidence indicates that both endogenous stress responses and exogenous corticosteroid therapy influence key biological processes involved in ocular homeostasis, including intraocular pressure regulation, inflammatory signaling, vascular permeability, and neurodegenerative pathways.

Glucocorticoids remain indispensable therapeutic agents for a wide range of inflammatory and immune-mediated ocular disorders. However, their clinical benefits must be balanced against well-recognized adverse effects, including steroid-induced ocular hypertension, glaucoma, cataract formation, delayed wound healing, and increased susceptibility to infection. Individual patient characteristics, treatment duration, route of administration, and underlying stress-related hormonal dysregulation may all contribute to variability in therapeutic response and complication risk.

The available literature also supports a clinically relevant role for psychosocial stress in several ocular conditions, particularly glaucoma, ocular surface disease, and central serous chorioretinopathy. Beyond its biological effects, psychological distress may influence disease perception, treatment adherence, and overall quality of life, highlighting the importance of a patient-centered approach to ocular care.

Collectively, these findings support an integrated model in which neuroendocrine, inflammatory, vascular, and behavioral factors interact to influence disease susceptibility, progression, and treatment outcomes. Recognizing stress as a potentially modifiable contributor to ocular disease reinforces the need for multidisciplinary management strategies that combine pharmacological treatment with individualized risk assessment, patient education, and psychosocial support.

### 4.1 Future directions

Future research should focus on clarifying the relative contributions of endogenous stress-related hormonal alterations and therapeutic glucocorticoid exposure to the development and progression of ocular disease. Well-designed prospective studies are needed to establish causal relationships and to identify factors associated with individual variability in treatment response and susceptibility to adverse effects.

Several areas warrant particular attention:

- Development and validation of stress-assessment tools specifically designed for ophthalmic populations.
- Investigation of chronobiological approaches to optimize the timing and effectiveness of corticosteroid therapy.
- Expansion of steroid-sparing and targeted immunomodulatory treatment strategies.
- Large-scale clinical evaluation of nanoparticle-based, sustained-release, and other advanced corticosteroid delivery systems.
- Implementation of multidisciplinary care models that integrate psychological support, patient education, and conventional ophthalmic management.

In parallel, further investigation of glucocorticoid receptor signaling, neuroinflammatory pathways, and stress-related vascular mechanisms may provide new therapeutic targets capable of preserving anti-inflammatory efficacy while minimizing treatment-related complications.

A more comprehensive understanding of the dynamic relationship between stress biology, glucocorticoid pharmacology, and ocular disease has the potential to advance personalized medicine in ophthalmology and ultimately improve long-term visual and patient-centered outcomes.
